# The Biosynthesized Zinc Oxide Nanoparticles’ Antiviral Activity in Combination with *Pelargonium zonale* Extract against the Human Corona 229E Virus

**DOI:** 10.3390/molecules27238362

**Published:** 2022-11-30

**Authors:** Abdulsalam A. Alqahtani, Mohamed A. El Raey, Eman Abdelsalam, Ammar M. Ibrahim, Omaish Alqahtani, Zenab Aly Torky, Hany G. Attia

**Affiliations:** 1 Department of Pharmaceutics, College of Pharmacy, Najran University, Najran 1988, Saudi Arabia; 2 Department of Phytochemistry and Plant Systematics, National Research Centre, Dokki, Cairo 12622, Egypt; 3 Department of Chemistry of Microbial and Natural Products, National Research Centre, Dokki, Cairo 12622, Egypt; 4 Applied Medical Sciences College, Najran University, Najran 1988, Saudi Arabia; 5 Department of Pharmacognosy, College of Pharmacy, Najran University, Najran 1988, Saudi Arabia; 6 Department of Microbiology, Faculty of Science, Ain Shams University, Cairo 11241, Egypt

**Keywords:** green synthesis, ZnONPs, *Pelargonium zonale*, antiviral activity, human coronaviruses

## Abstract

Almost one-third of all infectious diseases are caused by viruses, and these diseases account for nearly 20% of all deaths globally. It is becoming increasingly clear that highly contagious viral infections pose a significant threat to global health and economy around the world. The need for innovative, affordable, and safe antiviral therapies is a must. Zinc oxide nanoparticles are novel materials of low toxicity and low cost and are known for their antiviral activity. The genus Pelargonium was previously reported for its antiviral and antimicrobial activity. In this work, *Pelargonium zonale* leaf extract chemical profile was studied via high-performance liquid chromatography (HPLC) and was used for the biosynthesis of zinc oxide nanoparticles. Furthermore, the antiviral activity of the combination of *P. zonale* extract and the biosynthesized nanoparticles of ZnO against the human corona 229E virus was investigated. Results revealed that ZnONPs had been biosynthesized with an average particle size of about 5.5 nm and characterized with UV, FTIR, TEM, XRD, and SEM. The antiviral activity showed significant activity and differences among the tested samples in favor of the combination of *P. zonale* extract and ZnONPs (ZnONPs/Ex). The lowest IC_50,_ 2.028 µg/mL, and the highest SI, 68.4 of ZnONPs/Ex, assert the highest antiviral activity of the combination against human coronavirus (229E).

## 1. Introduction

World Health Organization (WHO) declared that the respiratory disease initiated in 2019 is Coronavirus Disease-2019 (COVID-19). In February 2020, after a month passed, WHO designated the worldwide extent of the disease as a pandemic [1].

The Coronaviridae family of the genus Coronavirus is part of the order Nidovirales. Due to their non-segmented, single-strand, positive-sense RNA genomes, Coronaviridae viruses are enveloped viruses. Infectious diseases caused by CoVs span a wide spectrum because of the viruses’ dual capacity to infect both people and animals [2]. There are seven types of coronaviruses, namely severe acute respiratory syndrome coronavirus (SARS-CoV), Middle East respiratory syndrome coronavirus (MERS-CoV), severe acute respiratory syndrome coronavirus 2 (SARS-CoV-2), 229E, NL63, OC43, and HKU1, are recognized to infect humans. Symptoms of these infections can range from those of the common cold to much more fatal diseases, like respiratory syndrome, as well as enteric and central nervous system diseases [3].

Human coronavirus-229E (HCoV-229E) is considered one of the major viral pathogens responsible for upper respiratory tract illness in humans. Up to now, there is no medicine for treating coronaviruses. In addition, there are a number of different vaccines, but there are new numbers of mutants that emerged, and hence there is an urgent need for new medications for coronaviruses [4].

Nanoscience is one of the emerging modern sciences which scientists have been paying attention to recently. This is a new field that includes the production and handling of nanoscale-size materials for several applications. Nanoparticles possess distinctive properties and exclusive characteristics such as shape, size, distribution, and chemical reactivity and have important applications in catalysis. Zinc oxide nanoparticles, on the other hand, were approved by FDA as GRAS materials. It is also used as an additive in animal feed like fish and pigs [5]. Fortunately, the physical processes of blocking virus attachment, infection, and uncoating have been identified as the mechanisms by which zinc-containing drugs exert their antiviral effects against various viruses. Moreover, Zinc is a vital element in our bodies that is found in body tissues like the brain, bone, muscles, and skin. Zinc is also a crucial component of various enzymes that are vital for metabolism, as well as nucleic acid and protein biosynthesis. ZnONPs are also absorbed by the body more easily than ordinary zinc compounds, so ZnONPs are expected to play an important role in fighting coronaviruses [6]. In addition, ZnONPs exhibit antiviral action against numerous viruses, including different respiratory viruses and herpes viruses, such as SARS-CoV-2 [7]. The plant kingdom is rich with plants that possess tremendous natural products that have diverse biological activities [8]. Previous in vitro and clinical research suggests that the root extract of the Pelargonium species has antiviral and immunomodulatory activities, which minimize the severity of the symptoms and the length of the illness caused by infections with a number of upper respiratory viruses [9]. Recently, the processes of green synthesis of ZnONPs based on plant extracts were found to be simple, fast processes, cost-effective, and, most importantly, clean, non-toxic, and eco-friendly. ZnONPs are greatly used for biomedical purposes as antibacterial, anti-diabetic, anti-inflammatory, anticancer, antiviral, anti-Alzheimer, drug delivery, and wound healing. It should be noted that the nature and type of the plant extract have an effect on the potentiality of the prepared metallic nanoparticles because it uses reduction and capping of the corresponding nanoparticles [10].

*P. zonale* belongs to the family Geraniaceae. It is often called a “geranium”, “zonale geranium”, or “zonale pelargonium”. Pelargonium leaves have a pleasant lemon aroma which is widely used as a flavoring agent in soaps, fruit desserts, ice cream, cake, and jellies formulations. *P. zonale* extract has several advantages: antioxidant activity, antimicrobial properties, and bacterial growth inhibition. An aqueous ethanol extract of *P. zonale* leaves was used as a reducing and stabilizing agent due to the presence of tannins, flavonoids, and lignans in the leaves that are responsible for the antioxidant capability of geranium, thus allowing its use for the synthesis of nanoparticles [11].

Our objective is to apply a green synthesis technique to synthesize the zinc oxide nanoparticles by reduction of zinc acetate using *P. zonale* extract and to study the antiviral activity of both the extract, ZnONPs, and their combination.

## 2. Results and Discussion

### 2.1. HPLC Investigation of Phenolic and Flavonoid Compounds of P. zonale Leaf Extract

**Figure 1:**
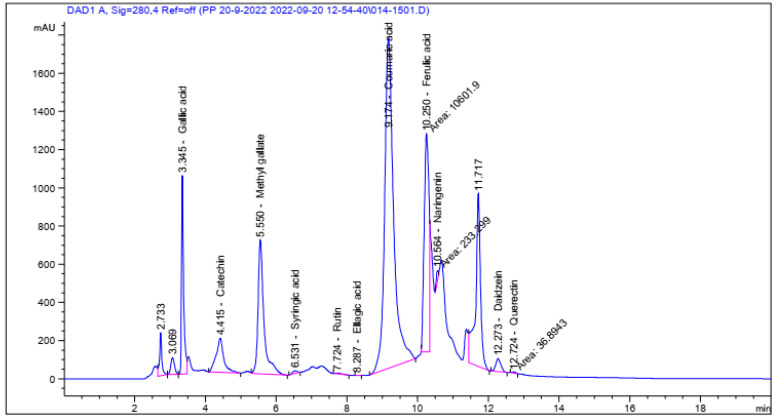
HPLC chromatogram of *P. zonale* leaves extract.

HPLC Investigation of Phenolics and Flavonoids (Figure 1) revealed the presence of eleven compounds in the *P. zonale* leaves extract. P-coumaric acid, catechin, ferulic acid, methyl gallate, and Gallic acid were identified as significant constituents at concentrations of 47.72, 39.99, 33.68, 27.69, and 19.90 mg/g, respectively (Table 1). The detection of catechin, gallic acid, methyl gallate, cinnamic acids, and quercetin was previously reported [12,13].

### 2.2. Synthesized ZnO Nanoparticles of P. zonale UV-Visible Characterization

Green-synthesized ZnO nanoparticles based on *P. zonale* leaf extract showed an absorption band at 320 nm representative of ZnONPs because of their high binding excitation energy and surface plasmon resonance (SPR) [14]. The remarkable blue shift absorption for the prepared ZnONPs can result from the presence of small particles. Another excitonic absorption band showed up at 270 and 285 nm (Figure S1). The absorption band that extends to longer wavelengths could be caused by the displacement of the electronic cloud on the entire skeleton of the ZnONPs.

### 2.3. Synthesized ZnO Nanoparticles of P. zonale TEM Characterization

**Figure 2:**
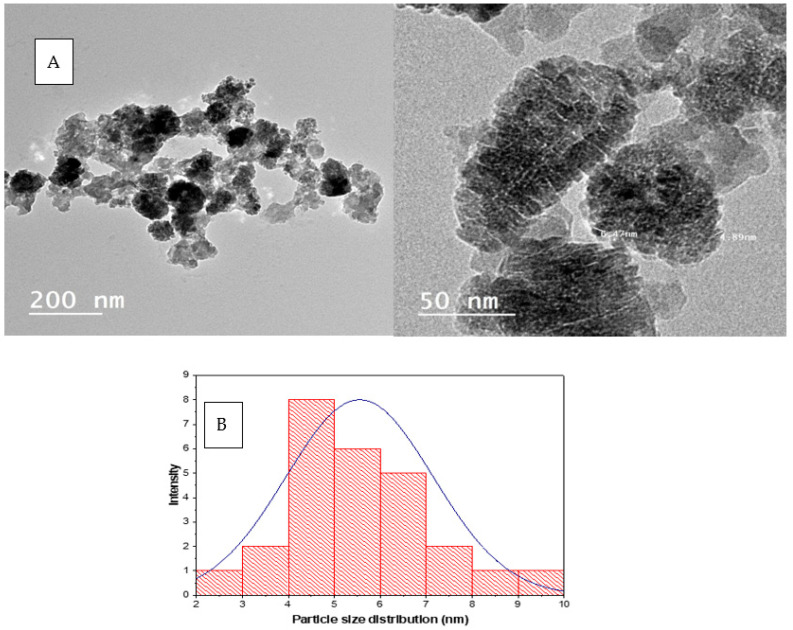
(**A**) TEM images of ZnO nanoparticles synthesized using *P. zonale* leaves extract (Pz-ZnO-NPs) and (**B**) particle size distribution histogram.

Transmission Electron Microscopy (TEM) image was used to describe the morphology and dimensions of the biosynthesized ZnONPs, as well as the size of the ZnONPs (Figure 2). TEM images displayed different figures, mainly irregular tiny spherical particles incorporated in big clusters, cubic structures, and rod-like shapes arranged like the Roman cluster, as illustrated by the TEM image. The particle size distribution histogram was 2–10 nm; this was derived from the TEM graph using the Image J software, and its Mean was 5.55 ± 1.6 nm.

### 2.4. FE-SEM and EDX Analysis

**Figure 3:**
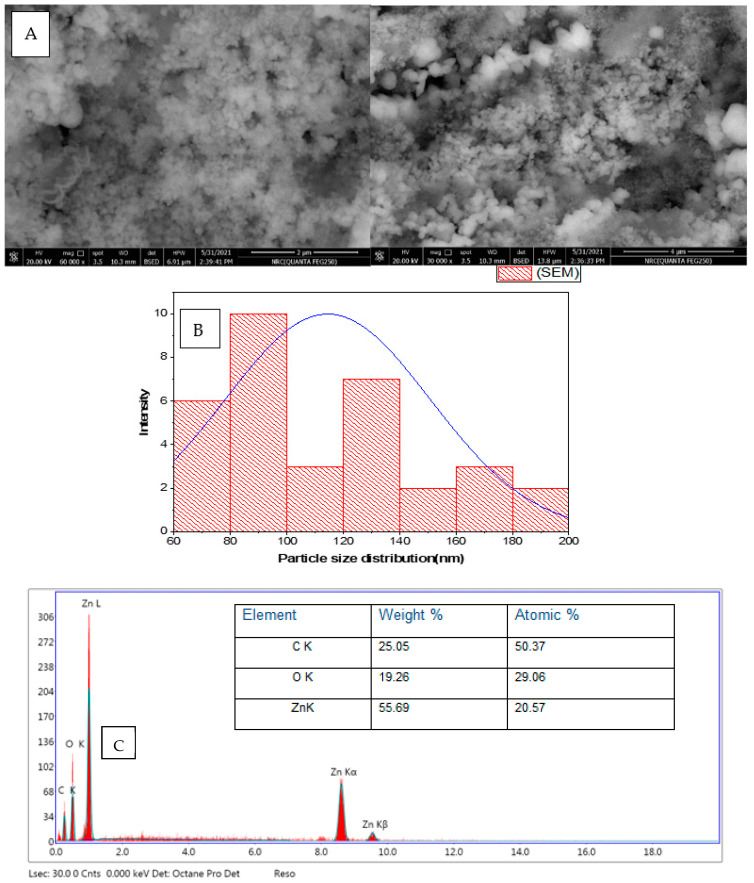
(**A**) SEM image *P. zonale* ZnO nanoparticles, (**B**) particle size distribution histogram (nm), (**C**) EDX spectrum of ZnO nanostructures synthesized using leaf extract of *P. zonale*.

The SEM image of *P. zonale* ZnO nanoparticles showed a spherical agglomerated n with a diameter was ranged from 65 to 200 nm with an average size of 114 nm Figure 3A.

The composition of the green synthesized ZnO nanoparticles (uncalcinated) was confirmed by EDX Figure 3C. Zn, O, and C elements were found in the ZnO nanoparticles to be 20.57%, 29.06 %, and 50.37% by atomic mass, respectively. The presence of C in the peak indicates *P. zonale*-containing compounds were used for mapping the prepared ZnO nanoparticles [15].

### 2.5. Analysis of X-ray Diffraction (XRD)

The crystal structure, shape, and size of the materials were investigated via X-ray diffraction. The positions of the peaks in the diffraction pattern specify knowledge about translational symmetry size and shape of the nanostructures. Thus, the green-synthesized ZnONPs powder of *P. zonale* leaves extract was inspected by X-ray diffraction to verify its crystalline form.

**Figure 4:**
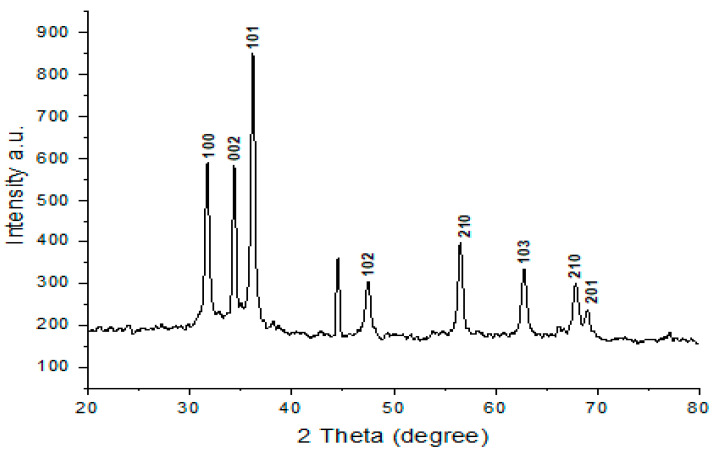
X-ray diffraction analysis (XRD) of the bio-synthesized ZnONPs using *P. zonale* leaves extract.

The XRD spectral analysis showed a number of strong diffraction peaks owing to Bragg reflections with 2θ values: 31.698° (100), 34.348° (002), 36.168° (101), 47.430° (102), 56.470° (210), 62.720° (103), 67.780° (212), 68.940° (201) Figure 4. These peaks agree with those described in the literature [16], suggesting that the produced nanoparticles are structurally identical to the zinc oxide phase described there. COD 2300112 [17].

The biosynthesized ZnONPs using *P. zonale* extract are crystalline, some with hexagonal wurtzite phase, and It is possible to assert that the obtained but contain some impurities which come from the capping agent as shown in EDX spectra (Figure 3C). The configurations are displayed in Figure 4. The average particle crystallite sizes were assessed using the Scherrer equation from the most intense peak at two theta (36.168), and FWHM (0.34) was found to be 5.66 nm).

### 2.6. Fourier Transform Infrared (FT-IR) Spectroscopy

**Figure 5:**
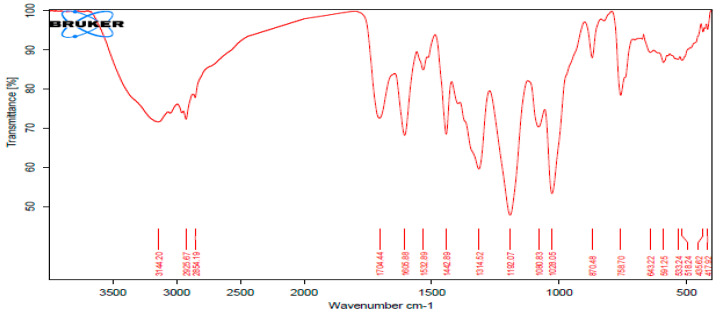
Fourier transform infrared (FT-IR) spectroscopy for *P. zonale* leaf extract.

**Figure 6:**
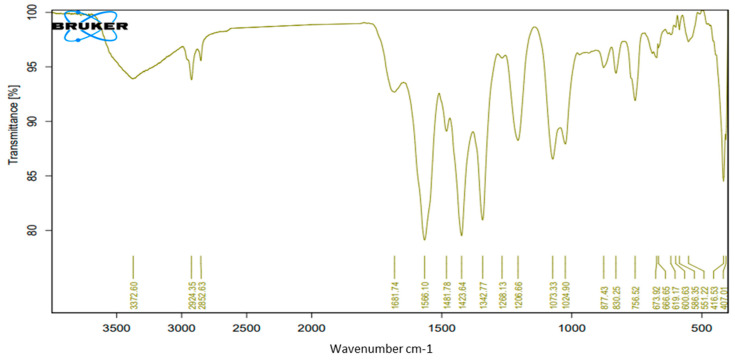
Fourier transform infrared (FT-IR) spectroscopy for ZnO nanoparticles based on *P. zonale* leaf extract.

FT-IR analysis is essential for identifying the functional groups on the surface of ZnONPs as well as their purity and composition; it also revealed the phytochemicals of the extract. The FT-IR spectral Figure 5 and Figure 6 show the main bands and key peak positions of the biosynthesized ZnONPs and extract: 3144.20 and 3372.31 cm^−1^, representing the broad peak of the OH stretching vibrations that verify the existence of phenolics compounds as capping agent on the surface of ZnONPs [18]. On the other hand, the peaks at 2925.67 and 2926.13 cm^−1^ can be attributed to the CH_3_ stretching vibration; 1605.88 and 1682.78 cm^−1^ point to the C=C bond stretching vibration.

The FT-IR spectrum verifies the presence of ZnONPs as a result of the presence of absorption peaks at 400 to 600 cm^−1^ [19,20]. The peak at 416.62 cm^−1^ can be attributed to the Zn–O stretching, thus confirming the synthesis of ZnONPs based on *P. zonale* leaves extract (Figure 5 and Figure 6).

The identified peaks in Figure 5 and Figure 6 indicate the existence of functional groups with strong hydrogen bonds, such as ketones, aldehydes, hydroxyls, alcohols, phenolics, and carboxylic acid compounds, which could be indicators of the presence of flavonoids, glycosides, and phenols; in addition to proving the synthesis of ZnONPs. Moreover, these results suggest that the *P. zonale* extract contains numerous metabolites (as also shown in Table 1) acting as capping and stabilizing agents for the biosynthesis of ZnONPs as well as constraining aggregation and unification of these nanoparticles. The reducing ability of plants is contingent on the presence of polyphenols and other chelating chemicals, which have a significant influence on the reduction potential of ions and the amount of generated nanoparticles. The likeness between the two spectra (Figure 5 and Figure 6) had some slight shifts in peak positions, indicating the presence of the residual *P. zonale* extract in ZnONPs as a capping agent.

### 2.7. Determination of the ZnO Nanoparticles Size by Zeta Sizer

**Figure 7:**
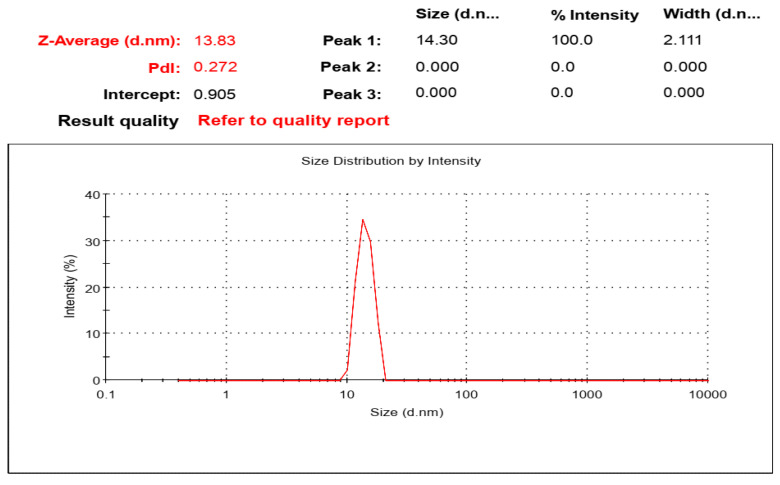
Zeta sizer of synthesized ZnO nanoparticles.

The particle size of prepared *P. zonale* ZnO nanoparticles was suspended at 70% aq. ethanol and measured by zeta sizer showing particle size equal to 13.83 ± 2.1 nm as shown in Figure 7. The particle size exceeded that determined by XRD and TEM due to the aggregation of some water molecules on the surface of ZnONPs, as shown by FT-IR spectra (Peak at 3327 cm^−1^).

### 2.8. Antiviral Activities of P. zonale (L.) Extract, Nano-ZnO Particles, and Their Combination

Viral infection and capacity investigated by In vitro antiviral assessment. Various health conditions, including viral infectious diseases, have been treated using plants in traditional medicine. There is a tremendous number of anti-infective and anti-tumor drugs from natural sources. Hence, in the near future, traditional medicinal herbs may prove to be powerful sources for obtaining novel antiviral drugs. The development of novel antiviral medications is a challenging mission because of their low toxic selectivity and the emergence of viral variations that are naturally resistant. Therefore, Viral resistance to antiviral medications is becoming more common, making it difficult to address viral disorders. Screening of plants as potential sources of antiviral leads resulted in the development of potent viral growth inhibitors and boosted the chance of discovering novel plant bioactive metabolites [21,22]. Applications of metal and metal oxide nanoparticles in virus-targeting formulations have demonstrated the outstanding diagnostic and therapeutic potential of the drugs, enhancing the functions of targeted medication delivery [23].

ZnONPs are considered promising scaffolds for creating antiviral medicines and nano vaccines due to their physicochemical properties. Blocking the virus’s entry into the cells and virostatic potential are the two most likely antiviral mechanistic mechanisms for ZnONPs [24,25]. Previously reported data states that Hesperidin and ZnONPs both exhibited antiviral efficacy against HAV, while ZnONPs displayed more activity [6]. Due to its ability to prevent virus entry, replication, and assembly, the ZnO and berberine combination can be considered viable anti-COVID-19 potential therapeutics. Additionally, it could be utilized to treat a secondary bacterial infection that developed in COVID-19 patients who were hospitalized. Furthermore, it was observed that the ZnO/berberine combination decreases the toxicity of long-term intake of hydroxychloroquine in vivo [26].

**Figure 8:**
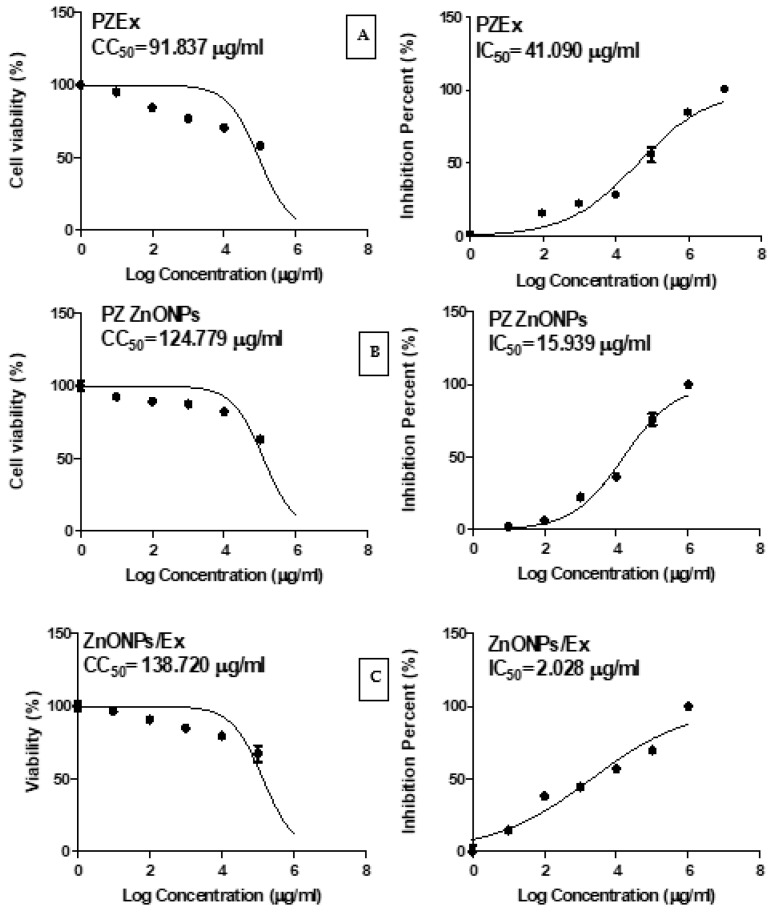
The cytotoxic effects (CC_50_) and the inhibition concentration (IC_50_) of the (**A**) PZ Ex, (**B**) PZ ZnONPs, and (**C**) PZEx/ZnONPs.

In this study, in vitro antiviral potential of *P. zonale* extract and biosynthesized nano-scaled ZnO particles were assessed against human coronavirus HCoV-229E. Cytopathic effect (CPE) assay was used to detect the activity of *P. zonale* extract (PZEx), ZnO nanoparticles (PZ ZnONPs), and their combination (ZnONPs/Ex). The results listed in Table 1 show significant activity and differences among the tested samples in favor of the combination of *P. zonale* extract (PZEx) and ZnONPs, and this result may be attributed to the role of ZnONPs that concurrently enhanced cell uptake and reduced cell efflux, Consequently, the intracellular drug concentration was effectively increased [27]. The CC_50_, IC_50_, and SI of these samples are shown in Figure 8 and listed in Table 2.

The viral inhibitory impact with IC_50_ of 41.090, 15.939, and 2.028 µg/mL, respectively, and CC_50_ of 91.837, 124.779, and 138.720 µg/mL, respectively, for *P. zonale* extract, ZnONPs, and their combination. It is clear that the IC_50_ of the combination of PZEx and ZnONPs increased to 2.028 µg/mL, revealing the higher activity of the combination. These findings proposed that the interaction between PZEx and ZnONPs was synergistic

Furthermore, the lowest IC_50_ 2.028 µg/mL and the highest SI 68.4 of ZnONPs/Ex assert the highest antiviral activity of the combination against human coronavirus (229E), while PZEx showed the lowest antiviral activity with SI 2.4. The results confirm the previously published data about how using ZnONPs actually reduce the infectivity of SARS-CoV-2 [28]. These findings demonstrated that the combination of PZEx and ZnONPs has greater antiviral activity than *P. zonale* extract and ZnONPs individually. Consequently, the tested samples are good candidates for further experiments as anti-coronaviruses.

## 3. Materials and Methods

### 3.1. Plant Material

*Pelargonium zonale* fresh leaves were collected during the summer of 2021 from an old Egyptian botanical garden called El-Orman located in Giza governorate, Egypt. The plant samples were recognized by botanist Mrs. Therris Labib and Dr. Mohamed El Gebali, specialists in plant taxonomy at El-Orman Botanical Garden. The plant parts were dried in the shade at a shade temperature. The dried parts were pulverized to a fine powder, then stored in a closed-colored glass container.

### 3.2. Preparation of the Plant Extract

The dried sample leaves (200 g) of *P. zonale* were extracted with 75% ethanol by maceration till exhaustion. The aqueous ethanolic extract of *P. zonale* was evaporated under low pressure using a rotavapor (Heidolph, Germany).

### 3.3. HPLC (High-Pressure Liquid Chromatography) Analysis

HPLC analysis was performed using an Agilent-1260 instrument. The separation was performed by an Eclipse C-18 column (4.6 mm × 250 mm i.d., 5 μm). The mobile phase included water plus 0.05% trifluoroacetic acid (A) and acetonitrile (B) at a flow rate of 0.9 mL/min. The mobile phase was undergone consecutively in a linear gradient as follows: 0 min (82% A); 0–5 min (80% A); 5–8 min (60% A); 8–12 min (60% A); 12–15 min (82% A); 15–16 min (82% A) and 16–20 (82% A). The multi-wavelength detector was adjusted at 280 nm. The injection volume was 5 μL for each of the sample solutions. The column temperature was kept at 40 °C.

### 3.4. Chemicals for ZnO Nanoparticles

Absolute ethanol, zinc acetate, and all the required materials were purchased of analytical grade from Elgomhoria Company, Egypt.

### 3.5. Biosynthesis of ZnO Nanoparticles by the Extract of Pelargonium Zonale

Synthesis of ZnO nanoparticles was performed by the method described by Alqahtani et al. 2022, in which 2 g of the dried *P. zonale* aq. Ethanolic extract was dissolved in 10 mL DMSO then a 500 mL solution containing 10 g of zinc acetate was added. The resulting mixture was stirred and reserved at 100 °C for half an hour, and the medium turned to alkaline by adding an ammonia solution dropwise. Whereby a yellow precipitate was formed. After 20 min, the zinc acetate had completely transformed into zinc oxide nanoparticles in the mixture. Finally, the resultant suspension was centrifuged for 10 min at 4000 rpm. The formed pellets were washed several times with distilled water and then absolute ethanol to remove any impurities. Finally, it was left to dry in ventilating hood [29].

### 3.6. Synthesized ZnO Nanoparticles (ZnONPs) Characterization

ZnONPs spectral properties were detected by UV Spectroscopy (UV spectrophotometer, Shimadzu UV-1601), Fourier transform infrared spectroscopy (FT-IR 6100 spectrometer, Bruker-Billerica, Billerica, MA, USA in the range of 4000–400 cm^−1^) for the functional group characterization that attached to ZnONPs surface, transmission electron microscope (TEM; JEOL model JEM 1011, Japan); in which drops of ZnONPs located on a grid of copper, covered with carbon support, and left at room temperature until dryness, The topography, and EDX of ZnO nanoparticles was investigated by SEM (Quanta FEG-250, FEL, Hillsboro, OR, USA). X-ray diffraction (XRD) of dry powders of the green-synthesized ZnONPs was accomplished with an X-ray diffractometer Bruker-D8 Advance Diffractometer (Bruker-AXS, Karlsruhe, Germany) with Cu Ka radiation (k = 1.54). Over a 2θ range of 10–90, the XRD pattern of ZnONPs was obtained using energy-dispersive x-ray spectroscopy to measure the XRD spectra, and the size of the most intense peak was measured using Scherrer’s equation. The nanoparticle size distribution was measured with a Zeta-sizer (Malvern instruments) in a disposable cell at 25 °C.

### 3.7. Evaluation of the Antiviral Activity

#### The Cytopathic Assay

Song et al. claim that the antiviral efficacy was assessed using the cytopathic inhibition effect (CPE) [30]. Cytopathic effect (CPE) inhibition assay will be used to identify potential antivirals against the human coronavirus (229E). The dose-response assay was designed to determine the range of efficacy for the chosen antiviral, i.e., the 50% inhibitory concentration (IC_50_), as well as the range of cytotoxicity (CC_50_). This assay is critical for determining antiviral efficacy in cell culture systems.

Nawah-Scientific, Egypt, provided the human coronavirus (229E) and Vero E6 cells. The DMEM media that the Vero E6 cells were cultured in was augmented with 10% fetal bovine serum and 0.1% antibiotic/antimycotic solution. Gibco BRL provided the antibiotic and antimycotic solution, fetal bovine serum, trypsin-EDTA, and DMEM medium (Grand Island, NY, USA). to evaluate the antiviral activity and cytotoxicity assays using the recently reported cytopathic (CPE) inhibition effect, Crystal violet method was used [31,32]. In brief, Vero E6 cells were seeded into a 96-well culture plate at a density of 2 × 104 cells/well one day before infection. The culture medium was removed the next day, and the cells were washed with phosphate-buffered saline. The infectivity of the Low Pathogenic Corona Virus (229E) was determined using the crystal violet method, which monitored CPE and allowed the percentage of cell viability to be calculated. 0.1 mL of diluted virus suspension of 229E containing CCID50 (1.0 × 10^6^) of virus stock was added to mammalian cells. This dose was selected to produce the desired CPEs two days after infection. For compound treatments, 0.01 mL of medium containing the desired compound concentration was added to the cells. Each test sample’s antiviral activity was determined using a ten-fold diluted concentration range of 0.1–1000 µg/mL: the virus controls (virus-infected, nondrug-treated cells) and cell controls (non-infected, nondrug-treated cells). For 72 hrs, culture plates were incubated at 37 °C in 5% CO_2_. The development of the cytopathic effect was monitored by light microscopy. Following a PBS wash, the cell monolayers were fixed and stained with a 0.03% crystal violet solution in 2% ethanol and 10% formalin. After washing and drying, the optical density of individual wells was quantified spectrophotometrically at 570/630 nm. The percentage of antiviral activities of the test’s compounds was calculated according to Pauwels et al. [33] using the following equation: antiviral activity= [(mean optical density of cell controls−mean optical density of virus controls)/(optical density of the test−mean optical density of virus controls)] ×100%. Based on these results, the 50% CPE inhibitory dose (IC_50_) was calculated. Before this assay, we assessed the cytotoxicity; cells were seeded at a density of 2x104 cells/well in a 96-well culture plate. The next day, The cells were incubated for 72 h in a culture medium containing serially diluted samples before being removed and washed with PBS. The subsequent stages were performed in the same manner as described previously for the antiviral activity assay.

The results of the 50% cytotoxic concentrations (CC_50_) and the 50% inhibitory concentration (IC_50_) were determined using GraphPad PRISM software (Graph-Pad Software, San Diego, CA, USA).

## 4. Conclusions

The increased prevalence of highly contagious viral diseases poses a severe threat to both human health and the global economy. The need for innovative, affordable, and safe antiviral therapies is urgent. Zinc oxide nanoparticles are low-cost and low-toxicity nanomaterial which is approved by FDA for safe use. *P. zonale* leaves extract chemical profile determined by HPLC, and it was capable of transforming zinc ions to ZnONPs with a size of about 5.5 nm, which was confirmed by different spectroscopic analyses (UV, FTIR, Tem, etc.). In this study, in vitro antiviral potential of *P. zonale*, ZnONPs, and their combination was assessed, enlightening the higher activity of the combination, and its lowest IC_50_ 2.028 µg/mL and the highest SI 68.4 assuring the potential antiviral activity of the combination against human coronavirus (229E). Further studies should be conducted on other different combinations of plant extracts and their biosynthesized nanoparticles, especially ZnONPs, to find innovative therapies as potential antiviral drugs.

## Figures and Tables

**Table 1:** Identification of Phenolic compounds of *P. zonale* leaves extract by HPLC.

	Compound	Area	Conc. (µg/g)	Conc. (mg/g)
1	Gallic acid	4380.80	19,895.28	19.90
2	Catechin	2672.75	39,993.08	39.99
3	Methyl gallate	8111.80	27,690.98	27.69
4	Syringic acid	126.07	582.47	0.58
5	Rutin	52.98	379.49	0.38
6	Ellagic acid	1.26	19.96	0.02
7	*p*-Coumaric acid	30,589.10	47,719.09	47.72
8	Ferulic acid	10,601.90	33,681.33	33.68
9	Naringenin	233.30	1336.70	1.34
10	Daidzein	685.42	2512.29	2.51
11	Quercetin	36.89	270.66	0.27

**Table 2:** The Anti-HCoV-229E activity of *P. zonale* extract, ZnONPs, and their combination.

Sample Code	CC_50_ (µg/mL) *	IC_50_ (µg/mL) *	SI *
PZEx	91.837	41.090	2.4
PZ ZnONPs	124.779	15.939	7.83
ZnONPs/Ex	138.720	2.028	68.4

* CC_50_, 50% cytotoxic concentration; IC_50_, 50% inhibition concentration; SI, selective index.

## Data Availability

The data is contained within the article and Supplementary Materials.

## Supplementary Materials

The following supporting information can be downloaded at: https://www.mdpi.com/article/10.3390/molecules27238362/s1, Figure S1: U.V analysis of *P. zonale* extract, PZ-ZnONPs, and PZ ZnoNPs/Ex, Figure 2S: IR Spectrum of *P. zonale* extract and PZ/ZnONPs.

## References

[1] Cucinotta D., Vanelli M. (2020). WHO declares COVID-19 a pandemic. Acta Biomed. Atenei Parm..

[2] Pal M., Berhanu G., Desalegn C., Kandi V. (2020). Severe acute respiratory syndrome coronavirus-2 (SARS-CoV-2): An update. Cureus.

[3] Desforges M., Le Coupanec A., Dubeau P., Bourgouin A., Lajoie L., Dubé M., Talbot P.J. (2019). Human coronaviruses and other respiratory viruses: Underestimated opportunistic pathogens of the central nervous system?. Viruses.

[4] Liu D.X., Liang J.Q., Fung T.S. (2021). Human coronavirus-229E,-OC43,-NL63, and-HKU1 (Coronaviridae). Encycl. Virol..

[5] Siddiqui S., Alrumman S.A. (2021). Influence of nanoparticles on food: An analytical assessment. J. King Saud Univ.-Sci..

[6] Attia G.H., Moemen Y.S., Youns M., Ibrahim A.M., Abdou R., El Raey M.A. (2021). Antiviral zinc oxide nanoparticles mediated by hesperidin and in silico comparison study between antiviral phenolics as anti-SARS-CoV-2. Colloids Surf. B Biointerfaces.

[7] Melk M.M., El-Hawary S.S., Melek F.R., Saleh D.O., Ali O.M., El Raey M.A., Selim N.M. (2021). Antiviral Activity of Zinc Oxide Nanoparticles Mediated by Plumbago indica L. Extract Against Herpes Simplex Virus Type 1 (HSV-1). Int. J. Nanomed..

[8] Attia G.H., Marrez D.A., Mohammed M.A., Albarqi H.A., Ibrahim A.M., Raey M.A.E. (2021). Synergistic Effect of Mandarin Peels and Hesperidin with Sodium Nitrite against Some Food Pathogen Microbes. Molecules.

[9] Papies J., Emanuel J., Heinemann N., Kulić Ž., Schroeder S., Tenner B., Lehner M.D., Seifert G., Müller M.A. (2021). Antiviral and Immunomodulatory Effects of Pelargonium sidoides DC. Root Extract EPs® 7630 in SARS-CoV-2-Infected Human Lung Cells. Front. Pharmacol..

[10] Faisal S., Jan H., Shah S.A., Shah S., Khan A., Akbar M.T., Rizwan M., Jan F., Wajidullah, Akhtar N. (2021). Green synthesis of zinc oxide (ZnO) nanoparticles using aqueous fruit extracts of Myristica fragrans: Their characterizations and biological and environmental applications. ACS Omega.

[11] Şöhretoğlu D., Sakar M.K., Sabuncuoğlu S.A., Özgüneş H., Sterner O. (2011). Polyphenolic constituents and antioxidant potential of Geranium stepporum Davis. Rec. Nat. Prod..

[12] Alqahtani A.A., Attia G.H., Elgamal A., Aleraky M., Youns M., Ibrahim A.M., Abdou R., Shaikh I.A., El Raey M.A. (2022). Cytotoxic Activity of Zinc Oxide Nanoparticles Mediated by Euphorbia Retusa. Crystals.

[13] Song J.-H., Choi H.-J., Song H.-H., Hong E.-H., Lee B.-R., Oh S.-R., Choi K., Yeo S.-G., Lee Y.-P., Cho S. (2014). Antiviral activity of ginsenosides against coxsackievirus B3, enterovirus 71, and human rhinovirus 3. J. Ginseng Res..

[14] Jayachandran A., Aswathy T.R., Nair A.S. (2021). Green synthesis and characterization of zinc oxide nanoparticles using Cayratia pedata leaf extract. Biochem. Biophys. Rep..

[15] Choi H.-J., Kim J.-H., Lee C.-H., Ahn Y.-J., Song J.-H., Baek S.-H., Kwon D.-H. (2009). Antiviral activity of quercetin 7-rhamnoside against porcine epidemic diarrhea virus. Antivir. Res..

[16] Lembo D., Swaminathan S., Donalisio M., Civra A., Pastero L., Aquilano D., Vavia P., Trotta F., Cavalli R. (2013). Encapsulation of Acyclovir in new carboxylated cyclodextrin-based nanosponges improves the agent’s antiviral efficacy. Int. J. Pharm..

[17] Pauwels R., Balzarini J., Schols D., Baba M., Desmyter J., Rosenberg I., Holy A., De Clercq E. (1988). Phosphonylmethoxyethyl purine derivatives, a new class of anti-human immunodeficiency virus agents. Antimicrob. Agents Chemother..

[18] Ganesh M., Lee S.G., Jayaprakash J., Mohankumar M., Jang H.T. (2019). Hydnocarpus alpina Wt extract mediated green synthesis of ZnO nanoparticle and screening of its anti-microbial, free radical scavenging, and photocatalytic activity. Biocatal. Agric. Biotechnol..

[19] Chen C.-C., Chen W.-C., Chiou M.-R., Chen S.-W., Chen Y.Y., Fan H.-J. (2011). Degradation of crystal violet by an FeGAC/H2O2 process. J. Hazard. Mater..

[20] Lu J., Batjikh I., Hurh J., Han Y., Ali H., Mathiyalagan R., Ling C., Ahn J.C., Yang D.C. (2019). Photocatalytic degradation of methylene blue using biosynthesized zinc oxide nanoparticles from bark extract of Kalopanax septemlobus. Optik.

[21] Iancu C., Cioancă O., Mircea C., Mocanu M., Hăncianu M. (2016). Pelargonium sp.: Characterization of the polyphenols and their biological potential. Farmacia.

[22] Hamed M., Mohamed M., Refai L., Hammam O., El-Ahwany E., Salah F., Hassanein H. (2015). The active constituents of Pelargonium zonale induced cytotoxicity in human hepatoma cell line HepG2. Int. J. Pharm. Appl..

[23] Kahsay M.H., Tadesse A., RamaDevi D., Belachew N., Basavaiah K. (2019). Green synthesis of zinc oxide nanostructures and investigation of their photocatalytic and bactericidal applications. RSC Adv..

[24] El-Hawwary S.S., Abd Almaksoud H.M., Saber F.R., Elimam H., Sayed A.M., El Raey M.A., Abdelmohsen U.R. (2021). Green-synthesized zinc oxide nanoparticles, anti-Alzheimer potential and the metabolic profiling of Sabal blackburniana grown in Egypt supported by molecular modelling. RSC Adv..

[25] Sowa H., Ahsbahs H. (2006). High-pressure X-ray investigation of zincite ZnO single crystals using diamond anvils with an improved shape. J. Appl. Crystallogr..

[26] Severson W.E., Shindo N., Sosa M., Fletcher Iii T., White E.L., Ananthan S., Jonsson C.B. (2007). Development and validation of a high-throughput screen for inhibitors of SARS CoV and its application in screening of a 100,000-compound library. J. Biomol. Screen..

[27] Li Q., Maddox C., Rasmussen L., Hobrath J.V., White L.E. (2009). Assay development and high-throughput antiviral drug screening against Bluetongue virus. Antivir. Res..

[28] Yadavalli T., Shukla D. (2017). Role of metal and metal oxide nanoparticles as diagnostic and therapeutic tools for highly prevalent viral infections. Nanomed. Nanotechnol. Biol. Med..

[29] Nasrollahzadeh M.S., Ghodsi R., Hadizadeh F., Maleki M., Mashreghi M., Poy D. (2022). Zinc Oxide Nanoparticles as a Potential Agent for Antiviral Drug Delivery Development: A Systematic Literature Review. Curr. Nanosci..

[30] Gurunathan S., Qasim M., Choi Y., Do J.T., Park C., Hong K., Kim J.-H., Song H. (2020). Antiviral potential of nanoparticles—can nanoparticles fight against coronaviruses?. Nanomaterials.

[31] Ghareeb D.A., Saleh S.R., Seadawy M.G., Nofal M.S., Abdulmalek S.A., Hassan S.F., Khedr S.M., AbdElwahab M.G., Sobhy A.A., Yassin A.M. (2021). Nanoparticles of ZnO/Berberine complex contract COVID-19 and respiratory co-bacterial infection in addition to elimination of hydroxychloroquine toxicity. J. Pharm. Investig..

[32] Liu J., Ma X., Jin S., Xue X., Zhang C., Wei T., Guo W., Liang X.-J. (2016). Zinc oxide nanoparticles as adjuvant to facilitate doxorubicin intracellular accumulation and visualize pH-responsive release for overcoming drug resistance. Mol. Pharm..

[33] Hosseini M., Behzadinasab S., Chin A.W.H., Poon L.L.M., Ducker W.A. (2021). Reduction of infectivity of SARS-CoV-2 by zinc oxide coatings. ACS Biomater. Sci. Eng..

