# Supplementary material for: The Biosynthesized Zinc Oxide Nanoparticles’ Antiviral Activity in Combination with Pelargonium zonale Extract against the Human Corona 229E Virus
Source: Molecules. 2022 Nov 30;27(23):8362. doi: 10.3390/molecules27238362 (PMC9736980; doi:10.3390/molecules27238362)
Supplement: Supplementary file 1 [file molecules-27-08362-s001.zip › molecules-2034916-supplementary.pdf]

Supplementary data

(Manuscript Ref. No.: Molecules-2034916)

"The biosynthesized zinc oxide nanoparticles' Antiviral activity in combination with *Pelargonium zonale* extract against the human corona 229E virus".

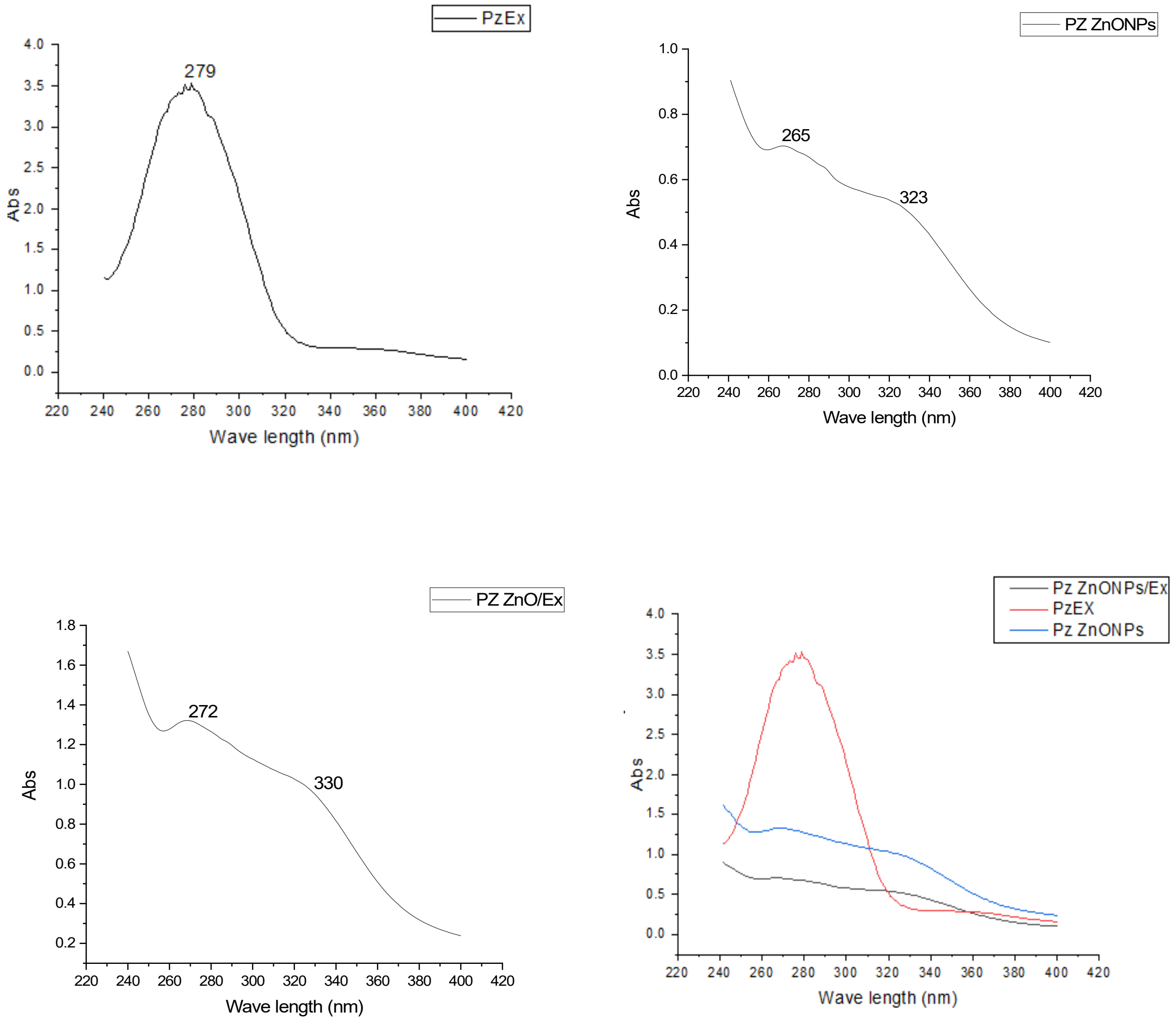

Figure S1. U.V analysis of *P. zonale* extract, PZ-ZnONPs, and PZ ZnoNPs/Ex.

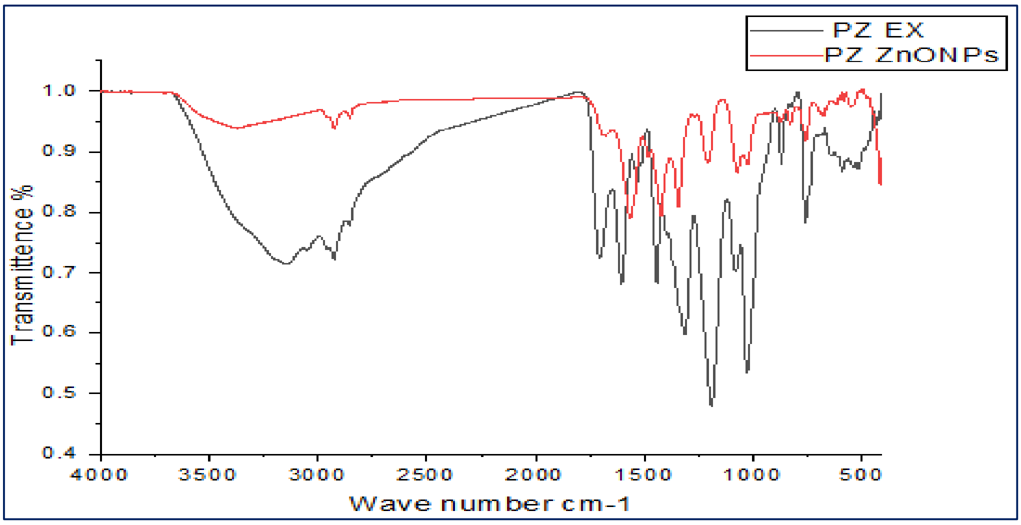

Figure S2. IR Spectrum of *P. zonale* extract and PZ/ZnONPs.
